# False Impressions

**Published:** 1888-11-03

**Authors:** Caroline Fothergill

**Affiliations:** Author of "An Enthusiast," "A Voice in the Wilderness," etc.


(*To be continued.*)

### FALSE IMPRESSIONS.

By CAROLINE FOTHERGILL, Author of "An Enthusiast," "A Voice in the Wilderness," etc.

( *Continued from p.* 64. )

### CHAPTER V.—WITH THE CHILDREN.

BEATRICE's belief that she would see a good deal of George at the Museum, was verified. She was constantly coming in contact with him, and found, to her annoyance, that Thursday evenings, on which she had her readings to the children, was also the evening on which he was used to come down and "superintend" the museum. It was seldom they did not meet; sometimes when the visitors were few and went away early, he would come into the room where Beatrice was and stand by the door a few minutes, listening and sending his keen, yet when he got among these outcasts of society, not unkind eyes, over the assembled multitude. He seldom made any remark when he came in, generally retiring before the reading was finished, and sometimes she did not see him again, and sometimes she did.

The first of these visits took place the very first time she went down to read. It was a bitterly cold night, which perhaps in some measure explained the unusually large audience which had gathered out of the streets into the warm, well-lit room. Fully a hundred children were present, and they presented a strange and instructive spectacle. Out of the hundred, perhaps half-a-dozen faces were clean, half-a-dozen suits or frocks in tolerable condition, half-a-dozen heads fairly smooth; the rest were ragged and dirty, and shock-headed to a degree which for a few minutes absolutely took away Beatrice's breath. It was difficult to read the faces, veiled under the thick coating of dirt, but most of the eyes were sparkling and keen, most of the attitudes expressed eagerness and expectation. Beatrice thought they looked ready for anything, and proceeded to act on her belief.

Few of the children were really ill-disposed, most of them were content to forego an hour's "larking," in consideration of the intellectual treat which was offered to them here. But there were a few who were distinctly "rowdy," and who would enjoy nothing more than to wreck the entertainment. These few required to be watched, and the policeman who was always in attendance at the building had offered to come up and keep his eye on the children while Miss Stanford read. Beatrice had declined his help; with the zeal of a novice, she was prepared to brave all dangers, and she held that the presence of a policeman would be degrading to the children, and irksome to herself. How were they ever to be civilized, if they were constantly confronted by force; and she resolved to manage them herself. That evening her choice of stories was unfortunate. She had not yet learned that to these inhabitants of the back streets and alleys of Mayfield, the most acceptable stories were those treating of ghosts, fairies, blood-curdling adventures, and hair-breadth escapes from apparently imminent death. The more utterly impossible they were, the better they liked them. A mere chronicle of childish doings had no attractions for them. No doubt this taste was the result of the monstrous confined life they led; but Beatrice had had no experience of such children, and it was only to be expected that at first she should make a mistake.

Her first story was listened to with respect and a tolerable amount of attention; and, although when it was over, and the children were told that those who were tired might go, numbers went out, yet equal numbers came surging in to replace them, and she began her second story. This did not go off so well. The children did not listen. They began to fidget, and then to talk in whispers; then to laugh and seize one another's caps, and throw them on the ground; after that to make remarks upon the reading.

"Eh, missis, read us somethin' else."

"Read us a ghost tale, missis."

"No, a fairy tale."

"Somethin' about birds, missis."

For some time Beatrice took no notice, except to mildly bid them "hush!" in which she was joined by a small minority of the audience, and to raise her voice a little. But the clamour increased. It soon became necessary to try to quell the tumult, for she could not hear herself speak. Her efforts were all in vain. Threats, and blandishments, and reasoning for the timebeing equally failed to make an impression. She could not still the din, and, at last, in despair she ceased reading and closed her book. A perfect babel of voices at once arose.

"Tell us a tale out o' your 'ead, missis."

"Read us somethin' wi' fire in it, about crocodiles an' that," demanded another; while a third remarked—

"Eh, missis, I am dry. Gi' me a sup out of your pitcher."

To this latter demand Beatrice weakly yielded. On the table at which she sat was a decanter of water with a tumbler placed there for her own use in case she got dry; but she yielded to the boy's petition for a "sup," and in an instant the whole crew was upon her, struggling for the water, tearing the glass from one another, surrounding herself, clinging to her, leaping upon her, until she could scarcely breathe.

While this tumult was going on, and she was fighting down a wish that she had accepted the policeman's proffered aid, the door opened, unheard by her, and she was unaware of any change having taken place, until suddenly, all in a moment the commotion ceased, perfect silence supervened; and, in looking up in amazement at this unexpected change, she saw George Murray standing just within the door.

He was looking at the scene before him with a smile upon his lips—not an unpleasant smile—as she had time to notice before his eyes met hers. Then his eyebrows rose, his lips curled upwards, and the cynical look she knew so well had covered the whole face.

She felt her colour rising as she got up from her chair. They had scarcely spoken since that first meeting here when the words of each had bristled with hidden meanings and stings, and now they met again under conditions the reverse of flattering to herself. She had had time to see how his first look of amusement at the scene before him had changed as soon as he saw who had charge of the children, and for an instant her mortification was swallowed up in the angry reflection which passed through her mind. Why was she always to appear to disadvantage before this man! There was time for no more before he came up to her saying—

"Good evening, Miss Stanford. I am afraid you have got more here than you can manage."

"Oh, not at all," she answered in what Laura would call "the Stanford voice." "The children were thirsty, and I was allowing them to drink. That is all."

"I understand," he answered rather drily, and she bit her lip as she reflected that he was probably perfectly well acquainted with this peculiarity of the children, and saw through her without any difficulty.

"Is the reading at an end?" he asked further.

They were now standing side by side; the children had withdrawn to their places where they now stood talking and laughing below their breath, and staring at the two who stood by the table from which the cloth had been dragged in the recent struggle for water.

Beatrice looked at her watch.

"I have not been here an hour yet," she said, "but I shall not read any more this evening. I think they have had enough."

"And yourself more than enough, probably; it is not often they behave like this? You are quite hoarse; let me dismiss them for you."

She made no answer, and he, without raising his voice in the least, apostrophized the crowd before him.

"Now, children, the reading is over. Thank Miss Stanford for her kindness in coming this evening, and go as quickly as you can."

The children looked at one another and giggled; there came some isolated cries of "Thank you missies," and then they clattered out of the room and downstairs.

"What were you reading to them?" he asked.

She handed him the book, and he turned over the leaves and said—

"These are capital tales for better educated children, but scarcely suitable for these."

She made no reply, not feeling the least inclination to explain the reason of her choice to him; and he went on talking for a little while, giving her hints as to managing the children, and so on. She did not hear him; she was feeling hot, tired, and disappointed, sore at heart, too. She had looked for a greater measure of success that evening, and it was particularly galling that her failure should have been witnessed by this man, of all men. Why could not another member of the committee have been there this evening?

She turned round suddenly and asked—

"Do you often come down here on Thursday evenings?"

"Always," he answered a little emphatically. "It has been my evening ever since the museum was opened."

She looked at her watch again and said—

"I think my cab will be here, and it is too cold to keep it waiting. Good-night."

She only bowed, without offering her hand. He opened the door for her and she went downstairs, meeting on the way the curator, who was coming to tell her that "her carriage had just arrived."

She said nothing of this meeting to Laura; she could not have borne Mrs. Ledward's comments, and she felt, too, as though it were only the beginning of sorrows.

George often came in to her readings, which it may be mentioned became a great success as she gained experience. Her manner and appearance could not fail to impress the children, and in a few weeks she had them completely under control. One evening she was rather late, and on reaching the room, looking very bright after her quick walk in the frosty air, she found the children all congregated round Mr. Murray, who was doing what they loved so much—telling them a tale out of his own head. It was a very marvellous tale. Beatrice heard a few sentences as she came to the door —enough to suggest that Mr. Murray had a very fertile imagination.

As soon as she came in he stopped, and apologised with a little ceremony for usurping her post, his excuse must be that the children had been growing restless; but he had not thought it was quite late enough to give her up altogether.

"You seem to be entertaining them very well," she said, making an effort to equal his indifferent tone—for she had come in a childrens' mood, and could not, all at once, lay it aside. "Please go on," she continued, it will do them no harm to have a change."

"Yes, yes," came from one or two of the children.

"I can listen too," she said. "No doubt I can learn a lesson from you; they are always wanting me to tell them tales out of my own head. Unfortunately I can't do it, I have no inventive gift."

"That is curious," was his reply, "As a rule women have more of it than men."

But he acceded to her wish, and finished the story-telling himself, Beatrice seated at the end of a bench, being not the least attentive of his audience.

In spite of this crack in the ice, they became no more friendly than before. Although they met almost every week, they had never yet shaken hands. Beatrice was cold and distant, George was polite, yet careless, as if the favour, or want of favour, which the well-born, well-dowered, and well-looking Miss Stanford should show him were alike indifferent to him. He never alluded to, or appeared to have the least recollection of their first two meetings; and Beatrice, who, in spite of Laura's advice, had intended in her queenly way to explain things to him, found herself for the first time in her life, tongue-tied by being confronted by a will stronger than her own. She had never undergone such an experience before, and she did not like it. When she was alone, the explanation seemed a most simple thing to make, but when she came face to face with George, and in the absence of all reference to the matter from him, felt the impossibility of speaking of it herself, her courage failed her, the words remained unsaid, and she was left feeling baffled, angry and disappointed.

She thought of it until the matter assumed an importance out of all proportion to its merits. She began to feel nervous in his presence, then to dread their meeting, at last to avoid him. And the more she turned from him, the more by some mysterious constitution of the human heart, she wished to stay, the more she thought he despised her, the more she desired his regard, and craved an opportunity of showing him she was better than he believed.

George's feelings may be seen from his corespondence with Dr. Temple. He had received a prompt reply to his letter describing his meeting with Beatrice and Mrs. Lennox. Dr. Temple refused to attach any weight to his friend's testimony. "Don't tell me," he wrote, "that a woman with Miss Stanford's face and voice and manner tells untruths. Its a farce on the surface of it. You have heard wrongly, or you have misunderstood what you heard. If you would only take the trouble to look for it, there will be some most simple and evident explanation of the 'mystery.'"

To this George had replied with brevity and comprehensiveness.

"You are an enthusiast, and it is no use arguing with you."

This was met by the counter accusation, "You are a morbid cynic. For Heaven's sake fall in love, and learn to believe in someone better than yourself. You are fast developing into a perfect specimen of the 'solemn prig' tribe. Save yourself while there is yet time, and don't write me any more about Miss Stanford till you can speak of her with decency and respect."

As it happened quite involuntarily, George did his friend's bidding. He was busy, and never answered the doctor's letter, and a change was destined before long to come over his opinion of Beatrice.

(*To be continued.*)

### COST OF A COTTAGE HOSPITAL.

The following accepted estimates for Falkirk Cottage Hospital, 10 beds, may be useful to others who are thinking of establishing a like institution: Brickwork, £119; joiner, £210; slater, £22 10*s.*; plasterer, £69 10*s.*; plumber, £85; total, £506. To the above sums there will fall to be added the following sums, viz.: Grates, stoves, &c., £20; painting, £40; furniture and furnishings, £164; surgical instruments, &c., £70; price of Salton Cottage and relative expenses, £300; architect's fees and contingencies, £50; making the total cost, £1,150.

